# Design of a sustainable supply chain network of biomass renewable energy in the case of disruption

**DOI:** 10.1038/s41598-024-64341-9

**Published:** 2024-06-10

**Authors:** Leila Aslani, Atefeh Hasan-Zadeh, Yousef Kazemzadeh, Amir-Hosein Sheikh-Azadi

**Affiliations:** 1 Fouman Faculty of Engineering, College of Engineering, University of Tehran, P.O. Box 4358139115, Fouman, Iran; 2 Department of Petroleum Engineering, Faculty of Petroleum, Gas, and Petrochemical Engineering, Persian Gulf University, Bushehr, Iran

**Keywords:** Biomass supply chain, Genetic algorithm, Simulated annealing algorithm, Stability, Disruption, Energy science and technology, Engineering, Chemical engineering, Energy infrastructure

## Abstract

Non-renewable energy sources, including fossil fuels, are a type of energy whose consumption rate far exceeds its natural production rate. Therefore, non-renewable resources will be exhausted if alternative energy is not fully developed, leading to an energy crisis in the near future. In this paper, a mathematical model has been proposed for the design of the biomass supply chain of field residues that includes several fields where residue is transferred to hubs after collecting the residue in the hub, the residue is transferred to reactors. In reactors, the residue is converted into gas, which is transferred to condenser and transformers, converted into electricity and sent to demand points through the network. In this paper, the criteria of stability and disturbance were considered, which have been less discussed in related research, and the purpose of the proposed model was to maximize the profit from the sale of energy, including the selling price minus the costs. Genetic algorithm (GA) and simulated annealing (SA) algorithm have been used to solve the model. Then, to prove the complexity of the problem, different and random examples have been presented in different dimensions of the problem. Also, the efficiency of the algorithm in small and large dimensions was proved by comparing GA and SA due to the low deviation of the solutions and the methods used have provided acceptable results suitable for all decision-makers. Also, the effectiveness of the algorithm in small and large dimensions is proven by comparing the genetic algorithm and simulated annealing, and the genetic algorithm's values are better, considering the deviation of 2.9%.and have provided solution methods suitable for all decision makers.

## Introduction

Renewable energy, which is also called reversible energy, refers to a type of energy whose production source, unlike non-renewable energy, can be renewed by nature during a short period of time or easily replaced after use. Recently, given that non-renewable energy sources are running out, these types of energy have been taken into consideration. In 2006, approximately 4% of the world's energy use was obtained from renewable energy, of which the share of biomass was approximately 14%, most of which was used for heating and the rest related to hydropower. The remaining 4% includes small hydropower plants, biomass, wind energy, solar energy, geothermal energy, and biofuels, which are increasing rapidly^1^.

Non-renewable energy sources, including fossil fuels, are a type of energy whose use far exceeds its natural production rate. Therefore, non-renewable sources will be exhausted if alternative energy is not fully developed, leading to an energy crisis shortly. To prepare for this inevitable reduction, the development of renewable energy becomes vital for human society. Renewable energy has various advantages such as sustainability, low emission of greenhouse gases and high economic efficiency. Among all these conventional types of renewable energy, biomethane has attracted increasing attention today. Therefore, it is widely used for home heating, electricity generation and vehicle fuel. It is estimated that one-fifth of the world's energy use is provided by methane^2^. The reactants for methane biogas production are usually derived from biowaste from animal manure, plants and crops and widely in fields. Almost inexhaustible raw materials for methane biogas determine its renewability and sustainability. In many cases, methane biogas production is less expensive than renewable energy sources. Because it uses biological waste. All these competitive advantages of methane biogas support it as an alternative energy in the future^3^.

**Figure 1:**
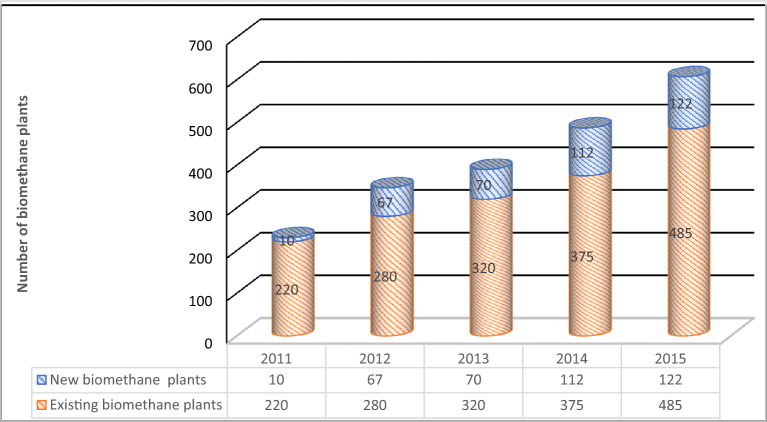
Biomethane plants in Europe.

**Figure 2:**
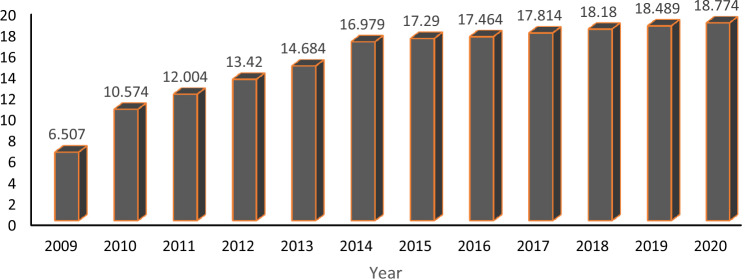
Evolution of the number of biogas plants in Europe.

Although the massive industrial production of methane biogas emerged at the beginning of this century; the method of methane biogas production has been popular in China and India for a long time^4^. Asian countries experienced a boom in methane biogas production at the end of the last century, and more than 78 biogas plants were built in Japan by 2012. The US methane biogas market is developing rapidly, with about 2000 methane biogas plants operating in the US by the end of this century reported that biogas production has increased in this country. As shown in Fig. 1, the EU has been encouraged by renewable energy policies to reach 18 billion cubic meters of methane in 2015, i.e., with more than half of the global biogas production, the European Union is currently the world leader^5^ (Fig. 2).

The biogas sector is expanding and has experienced major improvements in the last decade in terms of efficiency (both physical and economic) due to research and innovation. While this expansion continues, it is however starting to stagnate. 2009 to 2014 was a period of rapid growth averaging 2.094 new plants per year with an impressive 4.067 new plants built between 2009 and 2010 alone (over a 60% growth rate). Since 2014, growth has slowed with an average of only 299 new plants per year and the most recent data, 2019–2020, was slightly below average with only 285 new plants added. This reduced growth for biogas plants can be partly explained by the larger growth in biomethane plants (indeed, several small biogas plants are being converted to biomethane production).

Focusing on the distribution of these plants within the Member States, it is clear that it is far from uniform, with 60% of the plants installed in Germany (11.269 of the 18.774 in Europe). Only two EU Member States, Germany and Italy, have more than 1.000 plants, but it is likely that France will join them in the next few years if it maintains its current growth rate (+ 53 plants) given that it has the third-highest number of plants in the EU^5^.

In addition, as in reported that in 2013, the total number of biogas plants in Europe was 14,572. Germany has the most biogas industries with 9035 power plants, which accounts for 62% of the power plants. The total number of power plants after Germany is for Italy (1391 power plants), Switzerland (620 power plants) and France (610 power plants). In 2013, seven hundred and sixty new plants were established compared to 2012, which in fact increased by 5.5% reported that total biogas production in 2013 was equivalent to 65,731 GWh with 24,419 GWh of electricity and 34,762 GWh of thermal energy^6^.

On the other hand, Renewable energies such as wind energy, solar energy, biomass and other renewable energy sources, due to their advantages such as economic efficiency, compatibility with the environment and easy access, have recently attracted attention and are used to produce electricity and clean fuels. For this reason, the modeling of the renewable energy supply chain has attracted the attention of researchers. In this paper, a sustainable supply chain of field waste biomass was designed in the case of disruption. This chain includes a series of agricultural fields, where the residues of these agricultural products are transferred to the hubs. After keeping the residues in the hubs, they are sent to the reactors. After performing a series of processes, energy is produced in the form of gas from the residues. Since energy in the form of gas is less efficient, this energy produced in the form of gas should be converted into liquid form. For this reason, we send this obtained energy to the condensers to turn it into a liquid and increase its efficiency. Disruption may occur in any part of the network and affect the performance of the supply chain. As the supply chain becomes more complex, its different parts become more vulnerable to disruption. To reduce the negative impact of disruption, risk reduction strategies are used, such as cross-connections in condensers.

## Literature review

In a paper, Rentizelas et al. (2009) proposed a decision support system for multi-biomass energy conversion applications. The objective of the above system was to support an investor with a complete evaluation of the investment in the exploitation of several local biomasses for three generation applications (electricity, heating and cooling), in a given area^7^. The objective of a research paper was to develop an effective supply chain network design model for biogas production through anaerobic digestion of biomass. In this regard, a mixed integer linear programming model has been developed to determine the most suitable locations for biogas plants and biomass storage^8^. In the meantime, Jabbarzadeh et al. (2016) stated that the design of a sustainable supply chain network is one of the most important new and expanding research fields that affects the performance of the supply chain. As a specific and practical example, we can point to the fact that increasing regulations for carbon and waste management are forcing plants to consider their supply chain for environmental and social purposes. However, due to climate or natural disasters, a combination of other factors cause the facilities and their communication factors to be interrupted from time to time^9^.

On the other hand, in a study by Zarghami and Zakeri (2017), the high use of fossil fuels, taking into account source limitations, environmental pollution and increasing demand, has reduced over time and caused various sectors, including industry and transportation, to switch to other renewable energy sources^10^. In another study, a multi-level planning model has been developed from the perspective of the life cycle for the shale gas supply chain system. A set of interactive leader–follower objectives with an emphasis on environmental, economic, and energy concerns were included in the synergy optimization process. Then, an improved multi-level interactive solution algorithm based on the degree of satisfaction was proposed to improve the computational efficiency^11^.

In another study, three technologies (ammonia fiber expansion (AFEX) or pretreatment, fast pyrolysis, and leaf*-*protein concentrate (LPC)) were evaluated for profitability. A techno-economic model of a facility that can include multiple technologies and products was developed by Excel to be used for economic and environmental evaluation of potential systems^12^. In a paper by Dyken et al., a linear mixed integer modeling approach has been proposed for the components in a biomass supply chain, including supply, processing, storage and demand of different types of biomass. Biomass models were developed by current gas, electricity and heat infrastructure models in an optimization model designed for planning energy systems with multiple energy carriers^13^.

In a paper, Bai (2017) reported a comprehensive mathematical model for biomass combustion in the framework of a one-dimensional model^14^. In a study, Babazadeh et al. (2017) stated that *Jatropha corcas* L. as a bio-energy product has received much attention due to its high oil content for biodiesel production, tolerance to multi-year drought, soil improvement, desertification reduction, rural development and environmental benefits^15^.

In a paper, the development of a dynamic integrated biomass supply analysis framework and logistics model was described to simulate collection, storage and transportation operations to supply agricultural biomass to a biorefinery. This model has been developed using an object-oriented high-level simulation language^16^. In another paper, a multi-criteria optimization problem of regional biomass supply chains has been proposed for converting biomass into energy by simultaneously maximizing economic performance and minimizing environmental and social problems. The energy supply chain model included the layers of agriculture, pre-processing, processing and distribution^17^.

Fattahi et al. (2018) focused on sustainable planning of mining supply chain network from mining sites to demand centers. They proposed a cost-effective multi-stage stochastic program in which strategic and strategic planning decisions were integrated. Another study by Fattahi et al. (2018) focused on the design and planning of a biofuel supply chain network from biomass, which proposed a cost-effective multi-stage program in which greenhouse gas emission was reduced and the social effect of the supply chain was considered^18^.

In another study, to reduce greenhouse gas emission caused by the increasing use of fossil fuels, renewable energies that have few harmful pollutants have been used, among various types of which biofuels are prioritized due to their liquid nature and ability to be produced from various sources of biomass^19^.

In another study, it has been stated that in the face of increasing concerns about the negative environmental impact caused by human and industrial activities, doctors and policy makers of the biomass industry are very interested in the green supply chain to reduce carbon emission from supply chain activities. There are many studies that model biomass supply chain and environmental effects^20^. From another point of view, it was stated that due to sustainability, less environmental pollution and high profitability, the number of methane gas production systems is growing, i.e. successful commercialization in an economic investment for renewable biological energy. This issue has been investigated in a study. In this study, a mixed integer mathematical model has been proposed for the optimal location of the raw material collection center and plants for the production of biomethane gas to minimize the total operating cost of this supply chain system for renewable energy^21^.

In another study, it was stated that the design of supply networks enabled by renewable chemical raw materials presents complications in terms of uncertain markets, several options of intermediate chemical composition and uncertain chemical conversion path^22^. In a paper, Mafakheri and Nasiri (2019) presented a comprehensive review and classification of the important literature on modeling biomass supply chain operations, while relating them to the broader strategic challenges and issues related to the design, planning and management of biomass supply chains^23^.

In another paper, it was stated that in moving towards carbon-free energy networks, maintaining a reliable energy source with greater efficiency and lower cost was one of the objectives of all energy supply chain networks. Meanwhile, a supply chain model has been extracted and the governing equations have been solved using GAMS to achieve an optimal solution^24^.

In another study, a new general method was proposed for the synthesis of flexible supply networks with a larger number of uncertain parameters. The proposed method includes several steps that gradually from a nominal supply network obtained from fixed values of unknown parameters led to supplier networks, including deviation in unknown parameters and greater flexibility^25^. In a study, Morato et al. (2019) developed a framework for BCP localization using an iterative process model in geographic information system (GIS). This model used spatial distribution of biomass and road network maps and through an iterative process, prioritized areas with high biomass availability and short distance to roads^26^.

In a paper, Lin (2020) considered social enterprises and environmental uncertainties in a biomass plant location-forest biofuel problem. For this purpose, GIS was used to determine the candidate locations for acquiring forest biomass and opening, and then they solved the problem by fuzzy multi-objective linear programming^27^. In a paper, Yavari and Zaker (2020) stated that the effects of disruption in both the supply chain and its infrastructure have not been investigated simultaneously in previous studies. In addition, the risks of disruption have environmental effects in addition to economic effects. For this purpose, in a case study on the perishable nature of products and the risks of disrupting supply chain networks and electricity networks, a green closed loop flexible design problem has been investigated^28^.

Gital Durmaz (2020) addressed the optimal design and planning of the biomass supply chain network, including the flow from poultry farms to biogas facilities. GIS and Analytic Hierarchical Process (AHP) were used to determine the candidate location of biogas facilities^29^.

In a study, Nunes (2020) reviewed the literature on biomass supply chain modeling. So far, studies on supply chain models focused on evaluating specific supply chain scenarios, usually for cost minimization^30^. In a study, Mahjoub et al. (2020), to propose a network model for the design of the bioenergy supply chain, studied the combined second generation (for example, jatropha, agricultural residues and animal manure) and third generation (for example, microalgae) of biomass^31^.

In another study, Gilani et al. (2020) have used biomass, especially sugarcane, as a renewable energy source to produce bioethanol and other biofuels. In this study, the design of a strong three-phase optimal supply chain network for the production of bioethanol from sugarcane has been discussed^32^.

Rahemi et al. (2020) proposed a dual-objective mixed exact linear programming model for the optimal design and planning of the bioethanol supply chain network considering the competition of raw materials and biomass in existing agricultural lands. In a study, they addressed the issue that biomass is becoming an increasingly widespread energy source^1^. Mahjoub et al. (2020), to develop a bioenergy supply chain network model, studied the second (agricultural residues and livestock manure) and the third (microalgae) generation of biomass. A multi-objective stable mathematical model has been developed in this study^31^.

In another study, the effect of biomass availability and biogas demand on the economic and environmental performance of a biogas supply chain was investigated. A mixed integer nonlinear programming model was proposed as multi-objective, then the problem was reformulated as a single-objective problem with a multi-scenario strategy solution by CvaR^33^. The objective of another study was to design and build a database (mapping) for Portugal, to identify the availability of land for the implementation of energy crops and find agricultural and forestry production areas (including residues) with potential for sustainable exploitation for energy. ARCGIS was used as GIS and introduced data on soil type, water requirement, and edafocal conditions in shape and checkerboard data types to evaluate areas of interest for biomass planting^34^.

In another study, it was suggested that the use of biomass to produce chemicals, energy products and materials is an important way for sustainable development. This perspective provides a brief overview of the use of biomass as a renewable resource, analyzes its evolution over the years in terms of motivations and societal issues, highlights key contributions, and emphasizes how remaining challenges require the contribution of all aspects of the chemical sciences^35^.

In another study, the sustainable biological supply chain has been discussed as the key to sustainable biofuel production. The objective of the model was to show the environmental effect for creating a sustainable supply chain of biofuel and biogas^36^. In another paper, it was stated that a sustainable, efficient, competitive and safe energy system should be created to achieve the European Union's goal of carbon reduction by 2050 and the climate goals of the Paris Agreement. This paper proposed a mix of sustainable renewable energy supply networks in the EU-27 and a step-by-step energy transition in the transport and power sectors, achieving a net carbon neutral target by 2050^37^.

In another paper, a general optimization model was proposed for the selection of fuel conversion technologies, capacities, biomass locations, and transportation logistics from forestry resource locations to sites and then to final markets. A mixed integer linear programming model was developed and implemented by GEMS using databases built in Excel^38^. In another study, a two-level biomass source routing problem has been investigated. Considering a predetermined supply of biomass sources, a mixed integer programming model has been developed to determine the best locations for biomass collection facilities and corresponding vehicle routes^39^.

In another paper, facility location and vehicle routing problem for biomass supply chains were proposed. A mixed integer programming model was developed that can obtain optimal decisions for small-scale samples^40^. In another paper, the challenges of transitioning from a fossil fuel-dependent to a bio-based economy and the implications for the production of food, biologicals, and other bio-based materials were discussed^41^. In another paper, thermochemical conversion processes have been investigated due to higher efficiency, lower cost and greater versatility to provide a wide range of energy, fuel and chemical options^42^.

In previous studies, the levels of the chain were short, and more innovative and precise methods were used to solve the problem, and less meta-heuristic algorithms were considered, which has been addressed in this paper.

Supply chain is an important network in the business infrastructure that helps the production and distribution in the companies that sell their products very effectively, which provides a great source of jobs for many people. This is an infrastructure chain that includes resources such as human, company, data, activity and technology, and as a result, the final products are delivered to the buyers. These steps include cases such as converting raw materials into products and moving these products from the warehouse to the customer's home. Turning these steps into reality is the responsibility of people who work in industries such as warehousing, shipping, manufacturing, assembly and manufacturing, and transportation^43^. Supply chain is considered as a mechanism to reduce the final cost of product production for the customer. Supply chain management is a provision in the supply chain that optimizes the company's production processes for operational efficiency^44^.

Green supply chain management (GSCM) involves the integration of environmentally friendly practices in the traditional supply chain to achieve sustainable development. To increase the social responsibility of the organization, it is necessary to change the traditional method of supply chain management. Be green and try to achieve the objectives of sustainable supply chain is the most important solution in this field.

GSCM implies considering environmental issues in the organization's traditional supply chain. It also includes product design, supply of raw materials, production process, delivery of the final product to the customer and management of the product after use and its shelf life. GSCM in summary is the process of using environmentally friendly inputs and transferring the inputs to outputs that can be modified or reused at the end of the life cycle^45^.

GSCM was introduced by the Michigan State University Industrial Research Association in 1996, which is n fact a new management model for environmental protection. GSCM from the perspective of the product life cycle includes all steps of raw materials, product design and manufacturing, product sales and transportation, product use and product recycling. By supply chain management and green technology, the company can reduce negative environmental impacts and achieve optimal use of sources and energy.

Greening the supply chain is the process of incorporating environmental criteria or considerations throughout the supply chain. GSCM integrates supply chain management with environmental requirements in all steps of product design, selection and supply. Greening the supply chain is the process of considering environmental criteria or considerations throughout the supply chain,^46^.

In the 1990s, along with the improvement in production capabilities, industry managers realized that the materials and services obtained from different suppliers have a significant effect on increasing the organization's capabilities to meet the needs of customers, which in turn has a double effect on the organization's focus and left supply bases and sourcing strategies. Also, the managers realized that simply producing a quality product, sufficient in proportion, actually providing products with the criteria of the customer (when, where, and how) and quality and cost caused new challenges^47^.

Srivastava defined that green supply chain integrates environmental considerations into supply chain management, including product design, raw material search and purchase, manufacturing process, final product delivery process to consumers, and the end of the product life cycle management^48^.

Although in supply chain literature, the concepts of sustainable supply chain management and GSCM are usually used interchangeably, in fact, these two concepts are slightly different from each other. Sustainable supply chain management includes economic dimensions and social and environmental sustainability. Therefore, the concept of sustainable supply chain management is broader than GSCM. In the past, the product life cycle included processes from the design phase to consumption. While using the environmental management approach, it includes the processes of obtaining raw materials, designing, manufacturing, using and recycling, reusing and forming a closed loop of material flow to reduce source use and reduce the harmful effects of the environment^49^.

The need to reduce dependence on oil on the one hand and society's awareness to protect the environment on the other hand has caused research to be directed towards biomass supply chain management. Biomass, the source of biofuels, includes plant and animal materials such as wood, crops, seaweed, residual materials from agricultural and forestry processes, and industrial, human and animal organic wastes, which due to quick access, abundant supply and high efficiency, it has become a competitive source of energy and as a suitable alternative to fossil fuels, and it has recently attracted public attention to reduce greenhouse gas emissions and reduce the problem of climate change^23,50–52^.

Biofuels are produced through many conversion technologies, such as biochemical conversion processes, etc. Along with fossil fuels, nuclear energy and other renewable energies, they play an important role in the energy consumption market. The United States of America is the largest producer of biofuels, followed by Brazil and China^53,54^.

Due to the specific characteristics of biomass, this particular type of supply chain differs from classical supply chains in several ways. Different components in the bio-mass supply chain include: harvest and collection, storage, transportation, pre-treatment and conversion, and classic optimization models include network design problems, scheduling, facility location, vehicle routing problems and technology selection problems. However, mathematical optimization methods, including programming and heuristic algorithms to solve it, have been significantly used in the field of biomass supply chain management, both in the classical mode and in the biomass supply chain. In addition, environmental and sustainable development issues have created uncertainties, challenges and opportunities in the biomass supply chain^23,50–55^.

In the literature, some comprehensive reviews on optimization methods and biomass supply chains have been studied in the last decade^23,50–54^. The authors have classified optimization models into two categories: deterministic optimization models and stochastic models. The authors have reviewed the reviewed articles in the following aspects: decision-making level, supply chain structure, modeling approaches, quantitative performance measurement, shared information, novelty and practical application, assumptions, limitations, and future work^56–64^.

However, some focus on the mathematical modeling aspect and ignore heuristic algorithms, which are an important component of optimization methods, and in some research, more meta-heuristic solution approaches have been discussed. In general, the problems of this field include integrated supply chain planning, production process optimization, network design problems, scheduling problems, and location problems, and in general, the models are classified into two categories: the first are performance evaluation models and the second are optimization models. These optimization models are further classified into deterministic optimization, stochastic optimization and multi-objective optimization^65–70^.

Based on current trends and policies aimed at decarbonizing energy systems, the conversion of biomass to bioenergy has the potential for rapid growth, but such growth depends on the development of efficient, sustainable and competitive biomass supply chains. As a result, the biomass supply chain has attracted the interest of a diverse group of researchers in academia, government, and industry. The results of the literature review show that there are potential research gaps and opportunities in six critical areas^62–64^: integration of uncertainty with the bio-mass supply chain, considering risk in supply chain models; examining multi-product supply systems, strengthening supply chain flexibility, using inventory control methods and wider use of machine learning and artificial intelligence. In the current research, the supply of multiple products, the strengthening of flexibility through complete disruption in the facility (for example, a parameter has been considered that estimates whether the hub is capable of providing services in the scenario in question), taking into account the lack of random certainty and finally the role of the hub in reducing costs has also been investigated as a secondary goal in providing a mathematical model for the sustainable supply chain network of biomass renewable energy.

## Methodology

In this section, first, the mathematical model of the problem including the sets, parameters, decision variables, mathematical model and constraints of the problem are discussed, then the problem solving method is analyzed and compared by GAMS and GA.

In this research, the main goal is to provide a mathematical model for the sustainable supply chain network of biomass renewable energy in the condition of disturbance. This mathematical model includes types of products, seasons, farms, reactors, and secondary and main conductors in the supply chain network, and the disturbance is considered in general. In addition, as one of the secondary goals, the effective role of hubs in reducing the cost of transportation is also It is investigated and modelled so that the network flows are transmitted through this system, and ultimately, the goal of this research is profit maximization.

Because mathematical models need to be checked under the condition of disturbance, and this check is done in a scenario-oriented way. In this mathematical model, according to whether the facilities are active or inactive, we have 2 scenarios, 50% of the facilities are active and 50% are inactive, in the second scenario, the place of the facility is changed so that if in the first scenario, facility number 1 is active and facility If number 2 is inactive, in the second scenario, facility number 1 is disabled and facility number 2 is activated.

### Mathematical model

#### Sets, parameters and decision making variables

- Sets:
  P: index of products
  T: index of periods
  S: index of scenarios
  F: index of fields
  H: index of hubs
  R: index of reactors
  C: index of condensers
  C': index of secondary condensers
  T': index of transformers
  D: index of demand points
- Parameters:
  ${\alpha }$: percentage of natural loss of residues
  j: sale price of electricity
  $\widehat{{\text{I}}_{\text{pft}}}$: maximum supply of product p at time t from field f
  ${\widetilde{\text{I}}}_{\text{dt}}$: maximum demand of demand point d at time t
  M: big number
  ${\text{E}}_{\text{pfh}}$: cost of trasnferring product p from field f to hub h
  ${\text{B}}_{\text{phr}}$: cost of transferring product p from hub h to reactor r
  ${\upbeta }_{\text{rc}}$: cost of transfer from reactor r to condenser c
  ${\uptheta }_{{{\rm cc}^{\prime}}}$: cost of transfer from condenser c to sub-condenser c'
  ${\upmu }_{\text{cd}}$: cost of transfer from condenser c to demand point d
  ${\text{CO}}_{{{\rm t}^{\prime}}\text{d}}$: cost of trasfer from transformer t' and demand point d
  ${\text{FE}}_{{{\rm c}^{\prime}}{{\rm t}^{\prime}}}$: cost of transfer from condenser c' to transformer t'
  ${\upsigma }_{\text{h}}$: cost of constructing hub
  ${\gamma }_{r}$: cost of constructing reactor
  ${\omega }_{c}$: cost of constructing condenser c
  ${\delta }_{rc}$: cost of piping between reactor r and condenser c
  ${\partial }_{{{\rm cc}^{\prime}}}$: cost of piping between condensers c and c'
  ${\Omega }_{{{\rm t}^{\prime}}}$: cost of constructing transformer t'
  $\Phi {\text{M}}_{{{\rm t}^{\prime}}}$: cost of equipping transformer t'
  $${\text{Cap}}_{\text{r}}$$: reactor r capacity
  ${\text{EP}}_{\text{rc}}^{\text{S}}$: zero and one parameter, if under scenario s the piping between r and c is active, is equal to one and otherwise zero
  ${\text{E}}_{\text{c}}^{\text{S}}$: zero and one parameter, if under scenario s the facility c is active, is equal to one and otherwise zero
  ${\text{EP}}_{{{\rm cc}^{\prime}}}^{\text{S}}$: zero and one parameter, if under scenario s the piping between c and c' is active, is equal to one and otherwise zero
  ${\text{E}}_{\text{h}}^{\text{S}}$: zero and one parameter, if under scenario s the facility h is active, is equal to one and otherwise zero
  ${\text{E}}_{\text{r}}^{\text{S}}$: zero and one parameter, if under scenario s the facility r is active, is equal to one and otherwise zero
  $\beta$: the amount of loss due to disruption
  ${PS}_{s}:$ scenario event probability.

Decision variables are variables whose values are uncertain and determined by solving the problem and are the output of the problem as follows:

${\text{X}}_{\text{pfht}}^{\text{S}}$: product p sent from field f to hub h at time t under scenario s

${\text{L}}_{\text{phrt}}^{\text{S}}$: product p that goes from hub h to reactor r at time t under scenario s

${\text{Q}}_{\text{rct}}^{\text{S}}$: energy that goes from reactor r to condenser c at time t under scenario s

${\text{G}}_{{{\rm cc}^{\prime}}\text{t}}^{\text{S}}$: energy that goes from condenser c to sub-condenser c' at time t under scenario s is G_cc't^S

${\text{O}}_{{{\rm c}^{\prime}}{{\rm t}^{\prime}}\text{t}}^{\text{S}}$: energy that goes condenser c to transformer t' at time t under scenario

${\text{Y}}_{{{\rm t}^{\prime}}\text{dt}}^{\text{S}}$ : electricity under scenario s from transformer t' to demand points during period t under scenario s

${\text{u}}_{\text{h}}$: zero and one variable, if hub h is constructed, is equal to one and otherwise zero

${\text{K}}_{\text{r}}$: zero and one variable, if the reactor r is constructed, is equal to one and otherwise zero

${\text{V}}_{\text{c}}$: zero and one if the condenser c is constructed, is equal to one and otherwise zero

${\text{A}}_{\text{rc}}$: zero and one variable, if piping between reactor r and condenser c is done,is equal to one and otherwise zero

${N}_{cc{\prime}}$: zero and one variable, if the piping between condensers c and c' has been done, is equal to one and otherwise zero

${W}_{t{\prime}}$: zero and one variable, if transformer t' is equipped, is equal to one and otherwise zero

${I}_{{t}{\prime}}$: zero and one variable, if transformer t' is constructed, is equal to one and otherwise zero.

#### Objective function and constraints of the problem

The objective function of the problem is given in Eq. (1):

1 $$Max{Z}_{1}=\sum\limits_{s}{PS}_{s}\left\{\sum\limits_{{t}^{\prime}=1}^{{T}^{\prime}}\sum\limits_{d=1}^{D}\sum\limits_{t=1}^{T}J{Y}_{{t}^{\prime}dt}^{S}-(\sum\limits_{p=1}^{P}\sum\limits_{f=1}^{F}\sum\limits_{h=1}^{H}\sum\limits_{t=1}^{T}{E}_{pft}{X}_{pftt}^{S}+\sum\limits_{p=1}^{P}\sum\limits_{f=1}^{F}\sum\limits_{r=1}^{R}\sum\limits_{t=1}^{T}{B}_{phr}{L}_{phrt}^{S}+\sum\limits_{{t}{\prime}=1}^{{T}^{\prime}}\sum\limits_{c=1}^{C}\sum\limits_{t=1}^{T}{B}_{rc}{Q}_{rct}^{S}+\sum\limits_{c=1}^{C}\sum\limits_{{c}^{\prime}=1}^{{C}^{\prime}}\sum\limits_{t=1}^{T}{\theta }_{c{c}^{\prime}}{G}_{c{c}^{\prime}t}^{S}+\sum\limits_{{c}^{\prime}=1}^{{C}^{\prime}}\sum\limits_{{t}^{\prime}=1}^{{T}^{\prime}}\sum\limits_{t=1}^{T}F{E}_{{c}^{\prime}{t}^{\prime}}{O}_{{c}^{\prime}t{t}^{\prime}}^{S}+\sum\limits_{{t}^{\prime}=1}^{{T}^{\prime}}\sum\limits_{d=1}^{D}\sum\limits_{t=1}^{T}C{O}_{{t}^{\prime}d}{Y}_{{t}^{\prime}dt}^{S}+\alpha \sum\limits_{h=1}^{H}{\sigma }_{h}{U}_{h}+\propto \sum\limits_{r=1}^{R}{\mu }_{r}{k}_{r}+\alpha \sum\limits_{c=1}^{C}{\omega }_{c}{V}_{c}+\alpha \sum\limits_{r=1}^{R}\sum\limits_{c=1}^{C}{\delta }_{rc}{A}_{rc}+\alpha \sum\limits_{c=1}^{C}\sum\limits_{{c}^{\prime}=1}^{{C}^{\prime}}{\partial }_{c{c}^{\prime}}{N}_{c{c}{\prime}}+\sum\limits_{{t}^{\prime}=1}^{{T}^{\prime}}\sum\limits_{d=1}^{D}{\Omega }_{d{t}^{\prime}}{I}_{d{t}^{\prime}}\right\}$$

And constraints of the problem are:

2 $${X}_{pft}^{S} \le {\widetilde{A}}_{pft}\quad \forall s,p,f,t$$

3 $$\sum\limits_{p=1}^{P}\sum\limits_{h=1}^{H}{L}_{phrt}^{S}\le {cap}_{r} \quad \forall s,r,t$$

4 $$\sum\limits_{h=1}^{F}{X}_{phrt}^{S}(1-\alpha )\ge \sum\limits_{h=1}^{R}{L}_{phrt }^{S}\quad \forall s,p,t$$

5 $$\sum\limits_{h=1}^{H}{L}_{phrt}^{S}(1-\alpha )\ge \sum\limits_{c=1}^{C}{Q}_{rct}^{S} \quad \forall s,r,t,p$$

6 $$\sum\limits_{r=1}^{R}{Q}_{rct}^{S}(1-\alpha )\ge \sum\limits_{{c}^{{^{\prime}}}=1}^{{c}^{{^{\prime}}}}{G}_{c{c}^{{^{\prime}}}t}^{S} \quad \forall s,c,t$$

7 $$\sum\limits_{{c}^{{^{\prime}}}=1}^{{c}^{{^{\prime}}}}{G}_{c{c}^{{^{\prime}}}t}^{S}(1-\alpha )\ge \sum\limits_{{c}^{{^{\prime}}}=1}^{{C}^{{^{\prime}}}}{O}_{{c}^{{^{\prime}}}t{t}^{{^{\prime}}}}^{S} \quad \forall s,{c}^{ },t,{t}^{{^{\prime}}}$$

8 $$\sum\limits_{{c}^{{^{\prime}}}=1}^{{c}^{{^{\prime}}}}{O}_{{c}^{{^{\prime}}}t{t}^{{^{\prime}}}}^{S}(1-\alpha )\ge \sum\limits_{d=1}^{D}{Y}_{{t}^{{^{\prime}}}dt}^{S} \quad \forall s,{t}^{{^{\prime}}},t$$

9 $${Q}_{rct}^{S}\le M{EP}_{rc}^{S}{A}_{rc} \quad \forall s,r,c,t$$

10 $${O}_{ct{^{\prime}}t}^{S}\le M{E}_{c}^{s}{V}_{c} \quad \forall s, c,t,t{^{\prime}}$$

11 $${G}_{c{c}^{{^{\prime}}}t}^{S}\le M{EP}_{c{c}^{{^{\prime}}}}^{S}{N}_{{cc}^{{^{\prime}}}t} \quad \forall s,{c}^{{^{\prime}}},c,t$$

12 $${L}_{phrt}^{S}\le M{E}_{h}^{s}{U}_{h }\quad \forall s,p,h,r,t$$

13 $${Q}_{rct}^{S}\le M{E}_{r}^{S}{\mu }_{r } \quad \forall s,r,c,t$$

14 $${Y}_{{t}^{{^{\prime}}}dt}^{S}\le M{W}_{{t}^{{^{\prime}}}} \quad \forall s,{t}^{{^{\prime}}},d,t$$

15 $${\sum }_{t{^{\prime}}=1}^{T{^{\prime}}}{Y}_{t{^{\prime}}dt}^{S}\le {\widetilde{I}}_{dt}\times M\quad \forall s,d,t$$

The objective function is generally profit maximization. To obtain profit, revenue should be subtracted from all available costs. The revenue is equal to electricity produced from the transformer to the demand points. Also, the costs include the cost of transferring the product from the field to the hub, the cost of transferring the product from the hub to the reactor, the cost of transferring from the reactor to the condenser, the cost of transferring from the condenser to the sub-condenser, the cost of transferring from the condenser to the demand points, the cost of transferring between the transformer and the demand point, the cost of transferring from the condenser to the transformer, the cost of constructing the reactor hub, condenser, the cost of piping between the reactor and the condenser and between the condensers, the cost of constructing the transformer, and the cost of equipping the transformer.

Equation (2) in this objective function is revenue of selling energy. Equation (3) represents the transfer cost from the farm to the hub. Equation (4) represents the cost of transferring from the hub to the reactor. Equation (5) represents the cost of transferring from the reactor to the condenser. Equation (6) represents the cost of transferring between condensers. Equation (7) represents the cost of transferring from the condenser to the transformer and Eq. (8) represents the cost of transferring from the transformer to demand points. The following equations represent the cost of construction and equipping, which are described as follows. Equation (9) represents the cost of constructing the hub. Equation (10) represents the cost of constructing the reactor, and Eq. (11) represents the cost of constructing the condenser. Since the materials are moved between the reactor and the condenser through the pipe, so Eq. (12) represents the cost of piping between the reactor and the condenser. Equation (13) represents the cost of piping between the condensers because the materials in the condensers are also moved through the pipe. Equation (14) represents the cost of constructing transformers, and finally, Eq. (15) represents equipping transformers.

### Complexity of the problem

**Figure 3:**
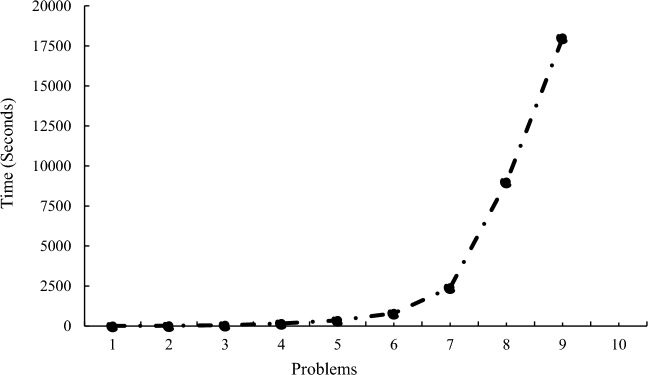
Significant increase in problem-solving time by GAMS.

To prove the complexity of the problem, different and random examples have been presented in different dimensions of the problem. As the dimensions of the problem increase, the time to solve the problem by GAMS increases exponentially, and then, the problem becomes unsolvable by GAMS as shown in Table 1 and Fig. 3. Accordingly, it can be said that the problem is complex and has no exact solution in large dimensions.

**Table 1:** Dimensions of the presented examples.

No	Set				
	F	D	R	C	H
1	5	3	2	2	2
2	6	3	2	2	2
3	7	3	2	2	2
4	8	4	3	3	3
5	9	4	3	3	3
6	10	4	3	3	3
7	12	5	4	4	4
8	14	5	4	4	4
9	15	5	4	4	4

### Efficiency of GA in small dimensions

In before section, it was proved that the problem is complex and it is necessary to use GA to solve the problem in large dimensions. For the accuracy and efficiency of the algorithm, five numerical examples in small dimensions were randomly generated and solved by GA, the results of which are given in Table 2 and then compared.

**Table 2:** Efficiency of GA in small dimensions.

No	Set					Objective function		
	F	H	R	D	C	GA	GAMS	Deviation (%)
1	6	2	2	3	2	15.02	15.1	0.67
2	8	3	3	4	2	22.97	23.3	1.30
3	10	3	3	5	2	30.1	30.5	1.67
4	11	4	4	6	3	33.5	34.3	2.39
5	12	4	4	7	3	40.8	42.2	2.93

According to the small difference in the average solution of GA compared to the solution of GAMS, it is proved that the presented GA has good efficiency in small dimensions. Table 2 shows the value of this deviation and the largest deviation is 2.93%, which is an acceptable value. Based on the valid references provided, the results of up to 3% difference are acceptable. For more information, you can refer to the references^71–79^.

**Figure 4:**
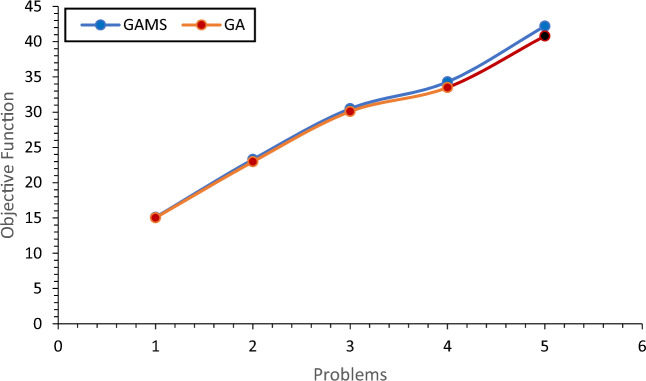
Comparison of the solution of GAMS with GA.

**Figure 5:**
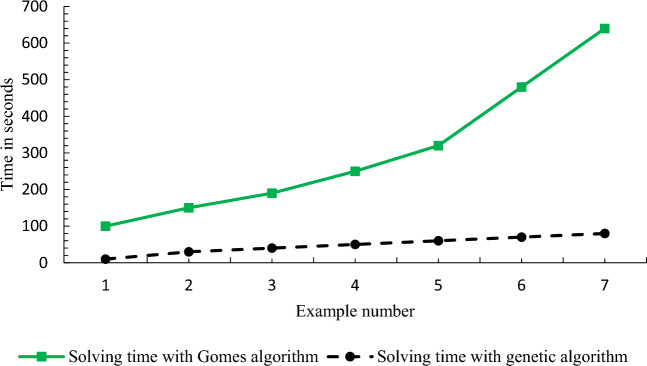
Comparison of problem-solving time by GA.

As shown in Fig. 4, the values of the objective function for both methods are almost close to each other, indicating the efficiency of the algorithm. Figure 5 shows the comparison of the solution time of the examples by GAMS and the algorithm used, indicating that problem-solving time of the algorithm is much shorter than that of GAMS.

### Efficiency of GA in large dimensions

In this section, the efficiency of the algorithm is investigated in two parts. First, the best solutions of GA are compared with the average solutions of GA. Second, solutions of GA will be compared with those of SA.

**Figure 6:**
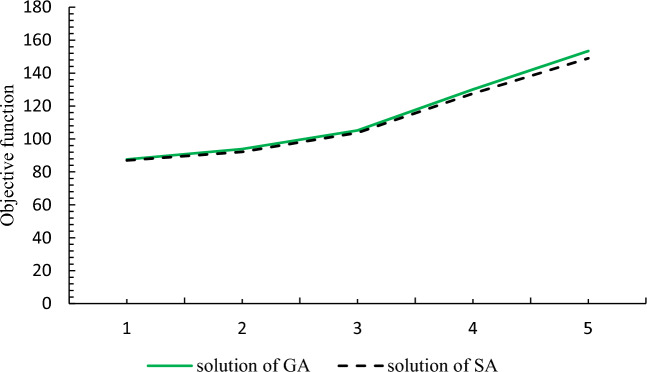
Efficiency of GA compared to SA.

For the performance of GA in large dimensions, some examples with random data in large dimensions have been presented, which were performed by GA for thirty times (the reason for solving the problem for thirty times is the normality of the population). In this case, the best solution obtained from solving the problem is compared with SA solutions as shown in Table 3 and Fig. 6. The comparison shows that the difference is very small and the maximum value in the objective function is 2.95, which proves the efficiency of GA in the large dimensions of the problem. In this section, several other examples in different dimensions are presented and compared by GA and SA.

**Table 3:** Eefficiency of GA compared to SA.

No	Number of sets					Objective function		
	F	C	R	D	H	Solution of SA	Solution of GA	Deviation (%)
1	15	4	3	10	3	87.02	87.6	0.69
2	25	5	4	12	3	92.9	93.9	0.97
3	40	7	5	15	4	103.8	105.2	1.35
4	60	8	6	20	4	127.6	130.1	1.95
5	80	10	7	25	5	149	153.4	2.95

### Comparison of problem-solving time by proposed methods

**Figure 7:**
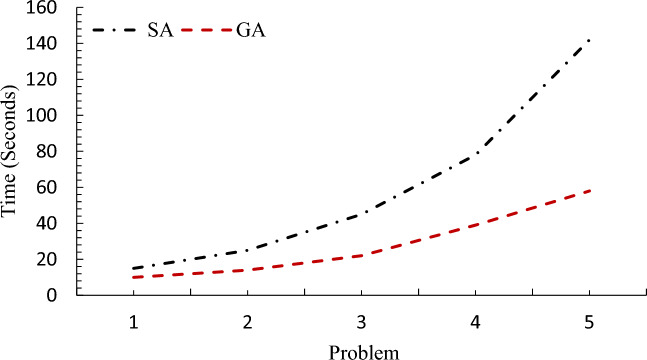
Comparison of problem-solving time by GA and SA.

**Figure 8:**
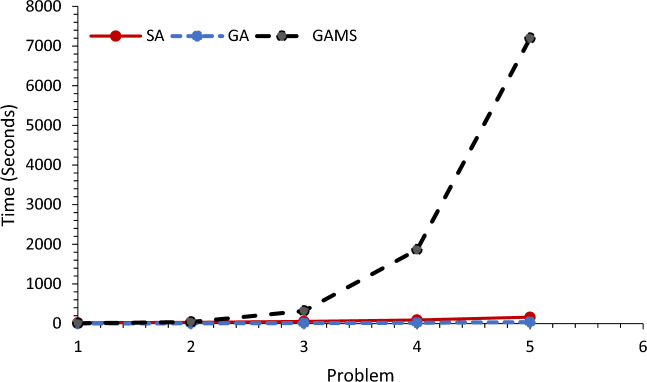
Comparison of problem-solving time by GA, SA and GAMS.

In this section, five random numerical examples in small and medium dimensions are presented and compared by GA, SA and GAMS. Table 4 shows the comparison of problem-solving time by the proposed method. As shown in Table 4 and Figs. 7 and 8, GA has less problem-solving time than the other two methods, followed by SA, and solving the problem by GAMS will take much more time.

**Table 4:** Comparison of problem-solving time by the proposed method.

Problem-solving time (s)			Set				
SA	GA	GAMS	L	S	H	D	F
15	10	8	2	2	2	3	5
25	14	42	2	2	3	4	8
45	22	321	2	2	3	5	10
78	39	1865	3	3	4	6	12
142	58	7201	3	3	4	7	15

One of the reasons why we solve mathematical problems with MATLAB software and algorithms like SA and GA is the high solution time by Gems software, which works very well in small dimensions and gives an accurate answer in an acceptable time. But in high dimensions, the time has greatly increased and this issue is inconvenient for researchers. According to the comparisons made between the three methods used to solve the mathematical model of this research and Table 4, Figs. 7 and 8 respectively show that the genetic solution time is better than the SA algorithm and both algorithms perform very well compared to Gems software.

For example, problem number 1 is 8 min for GAMS and 10 and 15 s for GA and SA, respectively, and for problem number 5, the comparison between solution methods based on the solution time metric for GAMS is very high and equal to 7201 s, and 58 and 142 were reported for other methods, respectively. This shows the importance of using meta-heuristic algorithms.

## Accuracy of the mathematical model

For the efficiency and accuracy of the mathematical model proposed in Chapter 3, a random numerical example was designed by MATLAB and then solved by GAMS. For the accuracy of the proposed model, the following two items should be checked.

- The constraints of the problem have been observed.
- The objective function values have been correctly calculated.

In this section, the number of demand points, condensers, and sub-condensers is equal to 3. There are 5 fields. The number of transformers, reactors, hubs, periods and products is 2, which is solved by GAMS. The problem data is attached.

According to the solution obtained from GAMS, the output of the problem is given in Tables 5, 6, 7 and 8. In the tables, it is specified which of the facilities has been constructed.

**Table 5:** Construction of reactors.

No	Level
1	Constructed
2	Constructed
3	–

**Table 6:** Construction of hubs.

No	Level
1	Constructed
2	Constructed

**Table 7:** Construction of transformers.

No	Level
1	Constructed
2	–

**Table 8:** Construction of condensers.

No	Level
1	–
2	–
3	Constructed

In this section, constraints of the problem are examined, and one of the constraint of the problem is the maximum constraint of supply for each field.

As shown in Tables 9 and 10, the maximum constraint of supply has been fully met. Another constraint of the problem is the reactor capacity, a sshown in Table 11. It has been proven that the capacity constraint of each reactor has been observed during each period.

**Table 9:** Maximum constraint of supply under scenario 1 (ton).

	Supply at t1 (ton)	Supply at t2 (ton)	Supply at t3 (ton)	Supply at t4 (ton)
F1	10	10	15	15
F2	25	25	16	16
F3	18	18	14	14
F4	14	14	17	17
F5	11	11	20	20

**Table 10:** Maximum constraint of supply under scenario 2 (ton).

	Supply at t1 (ton)	Supply at t2 (ton)	Supply at t3 (ton)	Supply at t4 (ton)
F1	10	10	15	15
F2	25	25	16	16
F3	18	18	14	14
F4	14	14	17	17
F5	11	11	20	20

**Table 11:** Reactor capacity constraint (ton).

No	Period	Demand (ton)	Capacity (ton)
1	1	19.6	80
1	2	26.6	80
2	1	89.6	110
2	2	88.2	110

Next, the value of the objective function based on the obtained solution is compared with the output of GEAM. As shown in Table 12, the calculated solution is the same as the output of GAMS. Therefore, the accuracy of the presented model is confirmed.

**Table 12:** Value of objective function ($).

Revenue ($)	430.2
Cost of transfer from field to hub ($)	120
Cost of transfer from hub to reactor ($)	123.4
Cost of transfer from reactor to condensor ($)	0.03
Cost of transfer between condensors ($)	0.03
Cost of transfer from condenser to transformer ($)	0.03
Cost of transfer from transformer to demand points ($)	0.08
Cost of hub construction ($)	0.03
Cost of reactor construction ($)	0.03
Cost of condenser construction ($)	0.03
Cost of transformer construction ($)	3
Total ($)	172.019

## Sensitivity analysis

In this section, for the sensitivity analysis of a random numerical example, different parameters of the problem and changes in the objective function of the problem have been investigated. In Table 13, the effect of increasing the value of the parameters on the value of the objective function is examined. In Table 13, for each of the parameters, the effect of changing the parameters is examined. In this table, the effect of increasing the parameters on the objective function has been investigated. For example, by increasing the parameter, the loss percentage of the objective function reduced.

**Table 13:** Sensitivity analysis of parameters.

Parameters	Effect on objective function
Percentage of loss	−
Selling price of electricity	+
Maximum of supply	+
Cost of transfer from field to hub	−
Cost of transfer from hub to reactor	−
Cost of transfer from reactor to condensor	−
Cost of transfer from condensor to sub-condensor	−
Cost of transfer from condensor to demand points	−
Cost of transfer between transformers and demand point	−
Cost of transfer from condenser to transformer	−
Cost of hub construction	−
Cost of reactor construction	−
Cost of transformer construction	−
Capacity of reactor	+

## Quantitative results

As shown in "Comparison of problem-solving time by proposed methods", the values of the objective function for both methods were almost close to each other, indicating the appropriate efficiency of the algorithm. Next, the solution times of the proposed examples by GAMS and the algorithm were compared, indicating that the algorithm's solution time was much shorter than GAMS solution. In Table 14, it has been proven that the efficiency of GA is small.

**Table 14:** Efficiency of GA in small dimensions.

No	Set					Objective function		
	F	H	R	D	C	GA	GAMS	Deviation (%)
1	6	2	2	3	2	15.02	15.1	0.67
2	8	3	3	4	2	22.97	23.3	1.30
3	10	3	3	5	2	30.1	30.5	1.67
4	11	4	4	6	3	33.5	34.3	2.39
5	12	4	4	7	3	40.8	42.2	2.93

In the next section, the efficiency of the algorithm is investigated in two parts. First, the best solutions of GA were compared with the average solutions obtained from GA. Second, GA solutions were compared with SA solutions. For the performance of GA in large dimensions, some examples with random data in large dimensions were presented, which were performed by GA for thirty times. In this case, the best solution obtained from solving the problem was compared with SA solutions. The comparison showed that the difference is very small and in the objective function, the maximum value was 2.98, which proved the proper efficiency of GA in the large dimensions of the problem. In this section, a number of other examples in different dimensions were presented and compared by GA and SA. In Table 15, the efficiency of GA algorithm compared to SA has been investigated and proven.

**Table 15:** Analysis of the efficiency of the GA compared to SA.

No	Set					Objective function		
	F	C	R	D	H	Solution of SA	Solution of GA	Deviation (%)
1	15	4	3	10	3	87.02	87.6	0.69
2	25	5	4	12	3	92.9	93.9	0.97
3	40	7	5	15	4	103.8	105.2	1.35
4	60	8	6	20	4	127.6	130.1	1.95
5	80	10	7	25	5	149	153.4	2.95

## Conclusion

Limited sources and environmental pollution caused by the use of fossil fuels and the increase in demand have made industry, transportation, etc. more likely to use other renewable energy sources. As a result, in recent years, the importance of designing, implementing and managing biological energy supply chains has increased. Therefore, in this paper, a sustainable supply chain of renewable energy was designed in the case of disruption. This supply chain consisted of agricultural land, hubs, reactors and condensers. What is important is the issue of disruption in the supply chain, which was discussed in this manuscript.

Also, first the required data was collected and a conceptual model was designed. After defining the conceptual model, a mathematical model was designed and solved by GAMS. To solve the problem in small dimensions, GA was proposed.

Among the limitations of this research, we can mention the lack of access to accurate data, as well as the atmosphere of uncertainty, which is always subject to changes, and managers always make decisions with risk, the result of which is a little far from reality. Also, the results show that the increase in demand, although it can be more profitable, on the other hand, it can put significant pressure on the supply system, and if the capacity of the facilities cannot bear it, it is necessary to apply resilient solutions or create new facilities to meet the needs of demand centres, which in any case will result in significant cost and time. These limitations cause managers to always use resilience strategies and have plans for the development of the supply chain to continue its activity and success.

Finally, for the accuracy of the model, GAMS was used and the efficiency of the algorithm was evaluated. In short, this study included the following objectives:

- Proposing a sustainable supply chain model for renewable energy by considering disruption and profit maximization
- Determining the supplied demand for each area and determining the amount of energy transferred at each level
- Determining the amount of product received from each part and field
- Proposing a mathematical model and investigating the accuracy of the model
- Proposing a meta-heuristic algorithm and investigating the efficiency of the algorithm
- Considering social and environmental goals separately
- Solving the mathematical model with exact methods like Bender decomposition

## Data Availability

All data generated or analyzed during this study are included in this published article.
